# Sleep and ageing: from human studies to rodent models

**DOI:** 10.1016/j.cophys.2020.03.004

**Published:** 2020-06

**Authors:** Laura E McKillop, Vladyslav V Vyazovskiy

**Affiliations:** Department of Physiology, Anatomy and Genetics, University of Oxford, Oxford, United Kingdom

## Abstract

Sleep duration and lifespan vary greatly across Animalia. Human studies have demonstrated that ageing reduces the ability to obtain deep restorative sleep, and this may play a causative role in the development of age-related neurodegenerative disorders. Animal models are widely used in sleep and ageing studies. Importantly, in contrast to human studies, evidence from laboratory rodents suggests that sleep duration is increased with ageing, while evidence for reduced sleep intensity and consolidation is inconsistent. Here we discuss two possible explanations for these species differences. First, methodological differences between studies in humans and laboratory rodents may prevent straightforward comparison. Second, the role of ecological factors, which have a profound influence on both ageing and sleep, must be taken into account. We propose that the dynamics of sleep across the lifespan reflect both age-dependent changes in the neurobiological substrates of sleep as well as the capacity to adapt to the environment.

**Current Opinion in Physiology** 2020, **15**:210–216

This review comes from a themed issue on **Physiology of sleep**

Edited by **Vladyslav Vyazovskiy** and **Jenny Morton**

For a complete overview see the Issue and the Editorial

Available online 16th March 2020

**https://doi.org/10.1016/j.cophys.2020.03.004**

2468-8673/© 2020 The Authors. Published by Elsevier Ltd. This is an open access article under the CC BY license (http://creativecommons.org/licenses/by/4.0/).

## Why and how do we age?

Ageing refers to a progressive deterioration of a broad range of physiological processes arising from structural and functional changes at the molecular, cellular and system levels. Some of these processes are physiological, while others reflect a progressive accumulation of unwanted consequences of various stressors. Many of the age-related changes are subtle and have little bearing on normal functioning, while others may have catastrophic consequences for physiology and behaviour, and may result in the development of disease and in some cases death. It is widely appreciated that animal models have important commonalities to humans, and therefore have been instrumental in investigating the mechanisms underlying ageing [1,2,3^•^].

There are a number of widely accepted hallmarks of ageing that can be subdivided into three categories [4,5^••^]. Firstly, primary hallmarks that are the causes of damage and include genomic instability [6], telomere shortening [7,8], epigenetic alterations [9,10] and cellular stress and altered proteostasis [11,12]. Secondly, antagonistic hallmarks of ageing that are the responses to damage and include de-regulated nutrient sensing via pathways such as IGF-1 signalling [13,14^•^], mitochondrial dysfunction (mitochondrial free radical theory of ageing) [15] and cellular senescence that is the arrest of the cell-cycle [16^•^,^17^]. Finally, integrative hallmarks of ageing that are the culprits of the phenotype of ageing (such as loss of organ function) and include stem cell exhaustion [5^••^] and altered intercellular communication (endocrine, neuroendocrine and neuronal) [18, 19, 20, 21, 22]. The neural mechanisms of ageing (both anatomical and functional) include a loss of synaptic connectivity and a decline in the function of specific brain circuits [19,23,24,25^•^]. These changes can have important consequences on overall behaviour and cognitive function [26].

Lifespan varies considerably among animal species and mammals in particular [27,28,29^••^]. Although it is still debated whether there is an upper limit of longevity, it is generally agreed that the probability of survival decreases with age in most species, indicating an existence of natural constrains on the life span [30,31]. It is thought that approximately 25% of the variation in longevity is due to genetic factors [32]. A number of evolutionarily conserved genetic pathways have been found to be involved in ageing (including genes related to endocrine signalling, stress responses, metabolism and telomeres) [14^•^,^33^], which allows the adaptability and plasticity of longevity to be investigated. However, it is also now widely accepted that extrinsic factors such as disease, predation, the timing of the activity period, the ability to fly and the foraging environment, can have significant influences on longevity, suggesting a remarkable plasticity of the ageing process [28,34].

Together these studies suggest that ageing is determined not only by genetic makeup, but also by environmental factors [32]. Furthering our understanding of the mechanisms underlying ageing has led to the development of a number of interventions that have successfully increased survival to old age in various species, including genetic and pharmacological manipulations [31,33,35], as well as caloric restriction [35, 36, 37, 38].

## Why and how do we sleep?

In all animal species waking and sleep alternate regularly, and in most cases spontaneous wakefulness never lasts for more than several hours continuously. It is believed that maintenance of waking and sleep states is regulated by the activity arising from several subcortical structures in the brainstem, hypothalamus and basal forebrain, which provide neuromodulatory (such as monoaminergic, glutamatergic, GABAergic and cholinergic) action on the forebrain [39, 40, 41, 42, 43]. Furthermore, sleep is regulated homeostatically [44,45], that is sleep loss is compensated by a subsequent increase in sleep duration and intensity. The best characterised physiological indicator of sleep-wake history is the level of cortical electroencephalogramm (EEG) slow-wave activity (SWA, 0.5–4.0 Hz) in non-rapid eye movement (NREM) sleep, which is high in early sleep and after sleep deprivation and decreases progressively to reach low levels in late sleep. The fundamental cellular phenomenon underlying sleep EEG slow waves is the slow oscillation, which is comprised of a depolarised UP state and a hyperpolarised DOWN state, during which the cortical cells cease firing [46].

Sleep characteristics (particularly sleep duration) vary greatly among the animal kingdom [47,48]. This may be the result of genetic variation, as it is widely accepted that sleep is under genetic control [49, 50, 51], and even within a species sleep can vary greatly depending on their genetic background [52]. For example, studies investigating sleep, circadian and light sensitivity have identified a number of important strain differences in mice [53, 54, 55]. Differences in sleep-wake characteristics are generally more stable within a species under controlled conditions, such as in the laboratory environment. However, notable differences may still remain. For example, under laboratory conditions factors such as food timing and availability, ambient temperature, lighting conditions or conditions favouring specific types of waking behaviours have a profound influence on various aspects of physiology, including sleep [56, 57, 58, 59, 60, 61]. Studies in animals in the wild have also revealed the importance of environmental variables, such as light, temperature and food availability on sleep [62^•^,^63^]. Perhaps not surprisingly, animals show pronounced differences in the amount or timing of sleep between captivity and the wild [64,65], and food availability or predation risk affect daily architecture of the rest-activity cycle [66].

Thus, sleep characteristics reflect an interaction between genetic factors and an individual’s experience that is their environment. Interindividual variation between animals provides an opportunity to investigate the function of sleep and the evolution of sleep strategies [62^•^]. It is possible that the differences in the amount of sleep and daily sleep-wake patterns observed across species (and often interpreted as differences in ‘sleep need’) may rather reflect adaptations to the environment.

## Sleep in ageing

It is well established that sleep undergoes fundamental and systematic changes across the lifespan, even in the absence of diagnostically identifiable pathology. In ‘healthy’ humans, sleep is deepest up until adolescence, after which it becomes progressively more fragmented and superficial, with sleep disorders becoming a growing issue in older individuals [67,68,69^••^]. The most notable effect of ageing is a decrease of EEG spectral power, particularly in the slow wave frequency range, and an attenuation of the homeostatic response to sleep deprivation observed as smaller rebounds in SWA after extended wakefulness [69^••^,^70^,^71^]. Several factors have been proposed to account for age-related sleep alterations in humans, including a reduced homeostatic sleep need, a reduced circadian drive, and/or a decreased capacity to generate and sustain consolidated sleep and the associated network oscillations [69^••^,^72^,^73^]. While the underlying mechanisms remain under investigated, the changes in sleep with ageing may be related to localised and diffuse structural brain changes, including cortical thinning of the prefrontal cortex and a degeneration of hypothalamic brain regions [67]. Interestingly, a recent study found an attenuation of transcriptome changes between sleep and sleep deprivation in the medial prefrontal cortex of old mice, and that sleep-active pathways such as DNA repair and synaptogenesis were particularly sensitive to the effects of ageing [74^•^].

Animal studies have been instrumental in investigating the mechanisms underlying sleep and ageing [2], with species such as worms and flies often utilised for their short lifespan and vertebrate models such as mice used for their genetic proximity to humans and a vast potential for transgenic engineering [53,55,75]. However, species can differ considerably with regards to their brain and body size, metabolic rates, and other aspects of physiology, which may have an impact on the association between sleep and ageing [76, 77, 78]. In addition, extrinsic factors such as the environment may also have dramatic effects on the association between sleep and ageing. For example, lighting conditions [79], diet [80] and exercise [81] have been shown to greatly affect this association in mice. Studies in rodent models partially replicated some aspects of human ageing such as an increase in the sleep-wake fragmentation and changes in the timing of sleep [12,82^•^,83^•^]. However, existing literature in rodent models is inconsistent, and notable differences between age-dependent changes in humans and rodents have been identified. In particular, recent evidence suggests that ageing in mice is instead associated with increased sleep duration and higher EEG SWA during NREM sleep [75,82^•^,83^•^,84^•^]. This has led to suggestions that in mice ageing may instead be associated with an increased homeostatic sleep need [82^•^,84^•^]. In addition, older mice may be less able to dissipate the increased sleep pressure, as shown by a slower decay rate of SWA during recovery sleep after sleep deprivation [84^•^]. These data are in direct contrast to data obtained from humans. This may be due to the shorter lifespan of rodents compared to humans [85], which may not be a realistic representation of ageing or disease time course. Mammals that are more closely related to humans may provide more translatable insights as to the effects of ageing on sleep in humans. Studies in non-human primates (NHP’s) are highly limited, perhaps due to the increased difficulty in performing such studies (both from technical and ethical points of view). Species such as sheep may however provide an important alternative to NHP’s though as their long lifespan and larger brains offer a number of advantages for studying human ageing and disease [86].

## Why have rodent and human studies on sleep led to conflicting results?

Historically sleep was viewed as a global, all-or-none phenomenon, although the existence of ‘partial sleep’ was postulated as early as the 1960s [87]. Recent studies in both humans and animals demonstrated that sleep may be initiated at the level of local cortical networks, with single neurons and local neuronal populations contributing to global sleep regulation [57,88, 89, 90, 91]. To this end, cortical regions may show different oscillatory activity depending on the region being recorded and the sleep-wake history of the individual, for which the term ‘local sleep’ was coined [90,92]. Clearly, both the global and local levels of sleep regulation are likely to be important for its overall functional role, yet their contributions to the overall sleep phenotype may be distinct [46].

Methodology for sleep recording and analysis matters in this regard. Although often considered the gold standard for sleep studies, conventional EEG recordings in humans have a poor spatial resolution and so local activities relevant for sleep regulation may not be easily detectable. This is a highly relevant limitation which should be considered when direct comparisons between humans and mice are made. Although evidence suggests that basic characteristics of brain oscillations are conserved across species [93], the recording technique is an important determinant of what exactly is recorded. Arguably, a single scalp EEG recording electrode conventionally used in humans, may record from a much larger area than the entire mouse brain. The discrepancies between humans and rodents may therefore be related to the level of organisation under scrutiny [94].

In our recent study we for the first time characterised the spatio-temporal properties of cortical neural activity across ageing in mice [82^•^]. We found that basic properties of spiking activity of cortical neurons, such as their average state-dependent firing rates, remain largely stable across the lifespan. This came as a surprise, given the pronounced changes in global sleep-wake architecture. Furthermore, local correlates of sleep homeostasis, such as characteristics of slow waves or associated neuronal population ON and OFF periods, were not markedly different between five-months (early adulthood) and two-year old (old age) mice. It is therefore unlikely that the observed changes in global sleep arise from a disruption occurring at the level of local cortical circuitry, and thus mechanisms underlying age-dependent changes in global sleep regulation are likely independent from mechanisms underpinning local sleep control [82^•^]. It remains to be established whether these findings can be generalised to humans.

It is possible that different species utilise different compensatory strategies in order to maintain cellular homeostasis and optimal functioning during wakefulness as an organism ages. For example, while mice increase the overall amount of sleep, humans may instead show an increase in the amount local sleep-like activity leaking into periods of wakefulness. Further studies are also required in order to determine whether indeed ageing in mice is associated with an increase in SWA or a redistribution of spectral power between frequencies and whether these effects can be generalised across the cortex. An intriguing possibility is that the progressive loss of synaptic connectivity or efficacy with ageing results in a ‘local deafferentation’, not dissimilar from the occurrence of sleep-like patterns of activity in the infarct lesion area in stroke patients [95], which allows cortical networks to remain in a local OFF state for longer periods. Together these data emphasise the importance of considering all levels of organisation (from local to global levels) together in order to understand the association between sleep and ageing.

**Figure 1:**
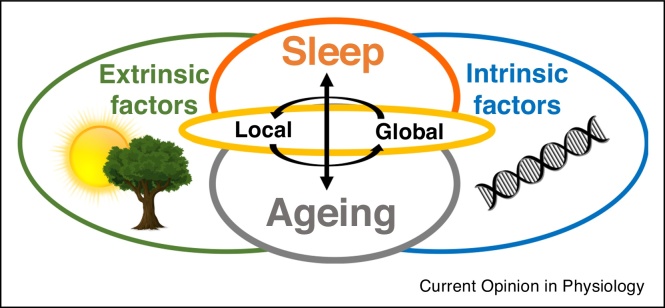
A schematic to demonstrate the complexity of the interaction between sleep (orange) and ageing (grey) and the important influences of both intrinsic factors (blue) such as genes and extrinsic factors (green) including the environment, specific waking experiences and ecological factors. Sleep undergoes progressive changes across the lifespan, which represents a combination of programmed ageing as well as the accumulation of damage resulting from environmental stressors. In addition, ageing influences the interaction of the organism with the environment, and thus it alters the role of ecological factors in sleep regulation. Sleep is a complex process regulated by local cellular/synaptic processes and global state control. Likewise, ageing manifests both at the level of local phenomena such as the occurrence of cellular stress or loss of synaptic connectivity as well as global changes in physiological functions and behaviour. Therefore both sleep and ageing must be considered at the various levels of organisation, from local to global mechanisms (yellow), as well as their interaction, in order to be fully understood.

Commonalities between animal models of ageing have enabled underlying mechanisms of ageing to be translated across species [3^•^]. However, the direct comparison between species may not always be straightforward, especially with regards to such complex phenotypes as sleep. For example, animal models of ageing are often selected based on them having early reproduction and short life spans, leading to an accelerated senescence and laboratory species are typically highly inbred [29^••^,^96^]. Ageing studies are now recognising the importance of characterising biological ageing rather than just capturing changes with the passage of time [97]. Mice have a much shorter lifespan compared to humans, with ∼70 years of age in humans corresponding to approximately 2 years of age in mice [85]. Therefore, species differences between rodents and humans may be related to differences in their biological age. Crucially, sleep and ageing are often studied in laboratory conditions fundamentally different from naturalistic conditions under which they evolved and their specific relevant traits (and corresponding genes) were selected. As previously discussed, sleep and ageing are under the influence of both genetic and extrinsic (e.g. environmental) factors. Laboratory conditions may present novel challenges (such as chronic stress induced by animals perceiving the environment as dangerous) that have not been previously experienced by species over evolutionary time [62^•^]. To this end, age-dependent changes in sleep in laboratory mice may represent an adaptation to chronic isolation, low and abnormally stable ambient temperature across 24 hours, lack of seasonal changes in the environmental variables or unrestricted food supply [62^•^,^98^]. Importantly, lifestyle and the environment also greatly affect ageing in humans [32]. For example, epigenetic markers (such as histone modifications) are well established to vary across the lifespan reflecting the experiences of individuals [2,9,10,99]. We therefore suggest (as depicted in Figure 1) that ageing and sleep reflect a complex interaction between intrinsic (such as genes) and extrinsic factors (such as the environment) and so the variation across species is expected and can only be fully understood when ecological context is taken into account.

## Development of interventions relevant for healthy and pathological ageing

In humans caloric restriction and exercise remain the most widely accepted best therapeutic interventions for improving symptoms of ageing [35]. Dietary components have been shown to be associated with inflammatory ageing (inflammageing), and personalised nutrition may have beneficial health effects potentially through the modulation of genetic and epigenetic factors [20]. The brain plays a central role in the regulation of ageing [19] and sleep [39], making it a promising target for intervention. Various pharmacological strategies have shown promising results in mice, such as epigenetic reprogramming [100], telomerase activators [16^•^] and the use of anti-inflammatory drugs [5^••^]. The elimination of senescent cells using senolytic drugs is also a useful target for diseases of ageing such as cancer and atherosclerosis, osteoarthritis and glaucoma, with beneficial effects on models of ageing and age-related disease noted [16^•^,^101^]. SASP-suppressing drugs (e.g. rapamycin; an inhibitor of the mTOR pathway) have also been shown to extend lifespan in various model organisms [16^•^,^102^]. It should however be noted that much of the success of pharmacological manipulations is based on studies in mice, and it remains largely unclear whether and to what extent cellular senescence contributes to ageing and related pathology in humans [16^•^].

Ageing is thought to be the biggest risk factor for neurodegenerative disease [19,103], and growing evidence has shown a high prevalence of sleep disturbances in neurodegenerative and psychiatric conditions [104]. This suggests that sleep disturbances may be both causally linked to, as well as sensitive to, brain pathology [105^•^]. There are a number of animal models available for studying neurodegenerative diseases, with their translatability reviewed elsewhere [105^•^,^106^,^107^]. In Alzheimer’s disease, the most common form of dementia, sleep disruptions are one of the earliest observable symptoms [70,108,109] but also may potentiate cognitive decline in such diseases of old age [70,110,111]. There are a number of theories as to the mechanisms that may underlie this association; including ineffective solute clearance via the glymphatic pathway and a dysfunction of the unfolded protein response, both of which have links to sleep regulation. Enhancement of SWA during sleep (for example using non-invasive stimulation techniques such as transcranial or acoustic stimulation) has been proposed as a novel treatment strategy for the renormalisation of cognitive functions [112, 113, 114, 115, 116]. Given the role of both intrinsic and extrinsic factors in age-related sleep alterations, as well as local and global aspects of sleep regulation (see Figure 1), it remains to be established what exactly needs to be enhanced in this context? If, as animal studies suggest, local mechanisms of slow-wave regulation are also intact in older humans, then the efforts should be directed not at enhancing slow waves as such, but at creating conditions to promote large-scale network synchronisation. To this end, animal studies remain instrumental for our understanding of the link between ageing, sleep and neurodegeneration.

## Conclusions

Here we outlined the insights gained from animal models as to the association between sleep and ageing, and highlight potential explanations for the species differences that have been observed. Firstly, we propose that only by considering all levels of organisation (i.e. from local cellular homeostasis to global network dynamics and peripheral mechanisms) will it be possible to further our understanding of the association between sleep and ageing. Secondly, we highlight the crucial importance of taking the ecological context into consideration as the differences in sleep between species may reflect the complex interaction of their specific evolutionary history and ecological and physiological demands [62^•^,^102^]. Finally, furthering our understanding of the mechanisms underlying sleep and ageing may allow for the identification of novel biomarkers of ageing and for the development of new interventions for age-related pathologies [70]. Given the complex nature of the association between ageing and neurodegeneration and the interaction with genetic and environmental factors, it is likely that a combination therapy consisting of both pharmacological and/or lifestyle modifications may be necessary in order for treatment strategies to be more successful [103].

## Conflict of interest statement

Nothing declared.

## References and recommended reading

Papers of particular interest, published within the period of review, have been highlighted as:

• of special interest
•• of outstanding interest

## References

[1] Castano-Prat P., Perez-Zabalza M., Perez-Mendez L., Escorihuela R.M., Sanchez-Vives M.V. (2017). Slow and fast neocortical oscillations in the senescence-accelerated mouse model SAMP8. Front Aging Neurosci.

[2] Sen P., Shah P.P., Nativio R., Berger S.L. (2016). Epigenetic mechanisms regulating longevity and aging. Cell.

[3•] Smith E.D. (2008). Quantitative evidence for conserved longevity pathways between divergent eukaryotic species. Genome Res.

[4] Aunan J.R., Watson M.M., Hagland H.R., Søreide K. (2016). Molecular and biological hallmarks of ageing. BJS Br J Surg.

[5••] López-Otín C., Blasco M.A., Partridge L., Serrano M., Kroemer G. (2013). The hallmarks of aging. Cell.

[6] Moskalev A.A. (2013). The role of DNA damage and repair in aging through the prism of Koch-like criteria. Ageing Res Rev.

[7] Blasco M.A. (2007). Telomere length, stem cells and aging. Nat Chem Biol.

[8] Young A.J. (2018). The role of telomeres in the mechanisms and evolution of life-history trade-offs and ageing. Philos Trans R Soc B Biol Sci.

[9] Steves C.J., Spector T.D., Jackson S.H.D. (2012). Ageing, genes, environment and epigenetics: what twin studies tell us now, and in the future. Age Ageing.

[10] Talens R.P. (2012). Epigenetic variation during the adult lifespan: cross-sectional and longitudinal data on monozygotic twin pairs. Aging Cell.

[11] Hafycz J.M., Naidoo N.N. (2019). Sleep, aging, and cellular health: aged-related changes in sleep and protein homeostasis converge in neurodegenerative diseases. Front Aging Neurosci.

[12] Naidoo N. (2018). Reduction of the molecular chaperone binding immunoglobulin protein (BiP) accentuates the effect of aging on sleep-wake behavior. Neurobiol Aging.

[13] Kanfi Y. (2012). The sirtuin SIRT6 regulates lifespan in male mice. Nature.

[14•] Kenyon C.J. (2010). The genetics of ageing. Nature.

[15] Hekimi S., Lapointe J., Wen Y. (2011). Taking a ‘good’ look at free radicals in the aging process. Trends Cell Biol.

[16•] de Magalhães J.P., Passos J.F. (2018). Stress, cell senescence and organismal ageing. Mech Ageing Dev.

[17] van Deursen J.M. (2014). The role of senescent cells in ageing. Nature.

[18] Laplante M., Sabatini D.M. (2012). mTOR signaling in growth control and disease. Cell.

[19] Satoh A., Imai S., Guarente L. (2017). The brain, sirtuins, and ageing. Nat Rev Neurosci.

[20] vel Szic K.S., Declerck K., Vidaković M., Vanden Berghe W. (2015). From inflammaging to healthy aging by dietary lifestyle choices: is epigenetics the key to personalized nutrition?. Clin Epigenet.

[21] Zhang G. (2013). Hypothalamic programming of systemic ageing involving IKK-β, NF-κB and GnRH. Nature.

[22] Zoncu R., Efeyan A., Sabatini D.M. (2011). mTOR: from growth signal integration to cancer, diabetes and ageing. Nat Rev Mol Cell Biol.

[23] Bishop N.A., Lu T., Yankner B.A. (2010). Neural mechanisms of ageing and cognitive decline. Nature.

[24] Morrison J.H., Baxter M.G. (2012). The aging cortical synapse: hallmarks and implications for cognitive decline. Nat Rev Neurosci.

[25•] Yeoman M., Scutt G., Faragher R. (2012). Insights into CNS ageing from animal models of senescence. Nat Rev Neurosci.

[26] Grady C. (2012). The cognitive neuroscience of ageing. Nat Rev Neurosci.

[27] de Magalhães J.P., Costa J., Church G.M. (2007). An analysis of the relationship between metabolism, developmental schedules, and longevity using phylogenetic independent contrasts. J Gerontol A Biol Sci Med Sci.

[28] Healy K. (2014). Ecology and mode-of-life explain lifespan variation in birds and mammals. Proc R Soc B Biol Sci.

[29••] Ricklefs R.E. (2010). Insights from comparative analyses of aging in birds and mammals. Aging Cell.

[30] Barbi E., Lagona F., Marsili M., Vaupel J.W., Wachter K.W. (2018). The plateau of human mortality: demography of longevity pioneers. Science.

[31] Dong X., Milholland B., Vijg J. (2016). Evidence for a limit to human lifespan. Nature.

[32] Passarino G., De Rango F., Montesanto A. (2016). Human longevity: genetics or lifestyle? It takes two to tango. Immun Ageing A.

[33] Kenyon C. (2005). The plasticity of aging: insights from long-lived mutants. Cell.

[34] Flatt T., Partridge L. (2018). Horizons in the evolution of aging. BMC Biol.

[35] Liang Y. (2018). Calorie restriction is the most reasonable anti-ageing intervention: a meta-analysis of survival curves. Sci Rep.

[36] Heilbronn L.K., Ravussin E. (2003). Calorie restriction and aging: review of the literature and implications for studies in humans. Am J Clin Nutr.

[37] Mattison J.A. (2017). Caloric restriction improves health and survival of rhesus monkeys. Nat Commun.

[38] Pifferi F. (2018). Caloric restriction increases lifespan but affects brain integrity in grey mouse lemur primates. Commun Biol.

[39] Eban-Rothschild A., Appelbaum L., de Lecea L. (2018). Neuronal mechanisms for sleep/wake regulation and modulatory drive. Neuropsychopharmacology.

[40] Liu D., Dan Y. (2019). A motor theory of sleep-wake control: arousal-action circuit. Annu Rev Neurosci.

[41] Ma S., Hangya B., Leonard C.S., Wisden W., Gundlach A.L. (2018). Dual-transmitter systems regulating arousal, attention, learning and memory. Neurosci Biobehav Rev.

[42] Scammell T.E., Arrigoni E., Lipton J.O. (2017). Neural circuitry of wakefulness and sleep. Neuron.

[43] Yamada R.G., Ueda H.R. (2019). Molecular mechanisms of REM sleep. Front Neurosci.

[44] Borbely A.A. (1982). A two process model of sleep regulation. Hum Neurobiol.

[45] Guillaumin M.C.C. (2018). Cortical region–specific sleep homeostasis in mice: effects of time of day and waking experience. Sleep.

[46] Vyazovskiy V.V., Harris K.D. (2013). Sleep and the single neuron: the role of global slow oscillations in individual cell rest. Nat Rev Neurosci.

[47] Anafi R.C., Kayser M.S., Raizen D.M. (2019). Exploring phylogeny to find the function of sleep. Nat Rev Neurosci.

[48] Campbell S.S., Tobler I. (1984). Animal sleep: a review of sleep duration across phylogeny. Neurosci Biobehav Rev.

[49] Funato H. (2016). Forward-genetics analysis of sleep in randomly mutagenized mice. Nature.

[50] Honda T. (2018). A single phosphorylation site of SIK3 regulates daily sleep amounts and sleep need in mice. Proc Natl Acad Sci U S A.

[51] Shi S., Ueda H.R. (2018). Ca2+ -dependent hyperpolarization pathways in sleep homeostasis and mental disorders. BioEssays News Rev Mol Cell Dev Biol.

[52] Franken P., Chollet D., Tafti M. (2001). The homeostatic regulation of sleep need is under genetic control. J Neurosci.

[53] Banks G. (2015). Genetic background influences age-related decline in visual and nonvisual retinal responses, circadian rhythms, and sleep. Neurobiol Aging.

[54] Eleftheriou B.E., Zolovick A.J., Elias M.F. (1975). Electroencephalographic changes with age in male mice. Gerontology.

[55] Hasan S., Dauvilliers Y., Mongrain V., Franken P., Tafti M. (2012). Age-related changes in sleep in inbred mice are genotype dependent. Neurobiol Aging.

[56] Eban-Rothschild A., Giardino W.J., de Lecea L. (2017). To sleep or not to sleep: neuronal and ecological insights. Curr Opin Neurobiol.

[57] Fisher S.P. (2016). Stereotypic wheel running decreases cortical activity in mice. Nat Commun.

[58] Ganeshan K., Chawla A. (2017). Warming the mouse to model human diseases. Nat Rev Endocrinol.

[59] Maloney S.K., Fuller A., Mitchell D., Gordon C., Overton J.M. (2014). Translating animal model research: does it matter that our rodents are cold?. Physiology (Bethesda Md).

[60] Northeast R.C. (2019). Sleep homeostasis during daytime food entrainment in mice. Sleep.

[61] Peirson S.N., Brown L.A., Pothecary C.A., Benson L.A., Fisk A.S. (2018). Light and the laboratory mouse. J Neurosci Methods.

[62•] Rattenborg N.C. (2017). Sleep research goes wild: new methods and approaches to investigate the ecology, evolution and functions of sleep. Philos Trans R Soc Lond B Biol Sci.

[63] Reinhardt K.D., Vyazovskiy V.V., Hernandez-Aguilar R.A., Imron M.A., Nekaris K.A.-I. (2019). Environment shapes sleep patterns in a wild nocturnal primate. Sci Rep.

[64] Daan S. (2011). Lab mice in the field: unorthodox daily activity and effects of a dysfunctional circadian clock allele. J Biol Rhythms.

[65] Rattenborg N.C. (2008). Sleeping outside the box: electroencephalographic measures of sleep in sloths inhabiting a rainforest. Biol Lett.

[66] van der Vinne V. (2019). Maximising survival by shifting the daily timing of activity. Ecol Lett.

[67] Baillet M., Schmidt C. (2020). Sleep, rest-activity fragmentation and structural brain changes related to the ageing process. Curr Opin Behav Sci.

[68] Gulia K.K., Kumar V.M. (2018). Sleep disorders in the elderly: a growing challenge. Psychogeriatrics.

[69••] Mander B.A., Winer J.R., Walker M.P. (2017). Sleep and human aging. Neuron.

[70] Muehlroth B.E., Werkle-Bergner M. (2020). Understanding the interplay of sleep and aging: Methodological challenges. Psychophysiology.

[71] Ohayon M.M., Carskadon M.A., Guilleminault C., Vitiello M.V. (2004). Meta-analysis of quantitative sleep parameters from childhood to old age in healthy individuals: developing normative sleep values across the human lifespan. Sleep.

[72] Cirelli C. (2012). Brain plasticity, sleep and aging. Gerontology.

[73] Klerman E.B., Dijk D.-J. (2008). Age-related reduction in the maximal capacity for sleep—implications for insomnia. Curr Biol.

[74•] Guo X. (2019). Age attenuates the transcriptional changes that occur with sleep in the medial prefrontal cortex. Aging Cell.

[75] Paulose J.K., Wang C., O’Hara B.F., Cassone V.M. (2019). The effects of aging on sleep parameters in a healthy, melatonin-competent mouse model. Nat Sci Sleep.

[76] Capellini I., Barton R.A., McNamara P., Preston B.T., Nunn C.L. (2008). Phylogenetic analysis of the ecology and evolution of mammalian sleep. Evol Int J Org Evol.

[77] Herculano-Houzel S. (2015). Decreasing sleep requirement with increasing numbers of neurons as a driver for bigger brains and bodies in mammalian evolution. Proc Biol Sci.

[78] Siegel J.M. (2005). Clues to the functions of mammalian sleep. Nat Lond.

[79] Panagiotou M., Deboer T. (2020). Effects of Chronic dim-light-at-night exposure on sleep in young and aged mice. Neuroscience.

[80] Panagiotou M., Deboer T. (2019). Chronic high-caloric diet accentuates age-induced sleep alterations in mice. Behav Brain Res.

[81] Panagiotou M., Papagiannopoulos K., Rohling J.H.T., Meijer J.H., Deboer T. (2018). How old is your brain? Slow-wave activity in non-rapid-eye-movement sleep as a marker of brain rejuvenation after long-term exercise in mice. Front Aging Neurosci.

[82•] McKillop L.E. (2018). Effects of aging on cortical neural dynamics and local sleep homeostasis in mice. J Neurosci.

[83•] Soltani S. (2019). Sleep–wake cycle in young and older mice. Front Syst Neurosci.

[84•] Panagiotou M., Vyazovskiy V.V., Meijer J.H., Deboer T. (2017). Differences in electroencephalographic non-rapid-eye movement sleep slow-wave characteristics between young and old mice. Sci Rep.

[85] Dutta S., Sengupta P. (2016). Men and mice: relating their ages. Life Sci.

[86] Perentos N. (2015). Translational neurophysiology in sheep: measuring sleep and neurological dysfunction in CLN5 batten disease affected sheep. Brain J Neurol.

[87] Hess R. (1964). The electroencephalogram in sleep. Electroencephalogr Clin Neurophysiol.

[88] Krueger J.M., Nguyen J.T., Dykstra-Aiello C.J., Taishi P. (2019). Local sleep. Sleep Med Rev.

[89] Siclari F., Tononi G. (2017). Local aspects of sleep and wakefulness. Curr Opin Neurobiol.

[90] Vyazovskiy V.V. (2011). Local sleep in awake rats. Nature.

[91] Watson B.O., Levenstein D., Greene J.P., Gelinas J.N., Buzsáki G. (2016). Network homeostasis and state dynamics of neocortical sleep. Neuron.

[92] Nir Y. (2011). Regional slow waves and spindles in human sleep. Neuron.

[93] Buzsáki G., Logothetis N., Singer W. (2013). Scaling brain size, keeping timing: evolutionary preservation of brain rhythms. Neuron.

[94] McKillop L.E., Vyazovskiy V.V., Landolt H.-P., Dijk D.-J. (2019). Sleep- and wake-like states in small networks in vivo and in vitro. Sleep-Wake Neurobiology and Pharmacology.

[95] Mensen A. (2019). Sleep as a model to understand neuroplasticity and recovery after stroke: observational, perturbational and interventional approaches. J Neurosci Methods.

[96] Miller R.A., Harper J.M., Dysko R.C., Durkee S.J., Austad S.N. (2002). Longer life spans and delayed maturation in wild-derived mice. Exp Biol Med.

[97] Jazwinski S.M., Kim S. (2019). Examination of the dimensions of biological age. Front Genet.

[98] Mason G.J. (2010). Species differences in responses to captivity: stress, welfare and the comparative method. Trends Ecol Evol.

[99] Snir S., Farrell C., Pellegrini M. (2019). Human epigenetic ageing is logarithmic with time across the entire lifespan. Epigenetics.

[100] Rando T.A., Chang H.Y. (2012). Aging, rejuvenation, and epigenetic reprogramming: resetting the aging clock. Cell.

[101] Childs B.G. (2017). Senescent cells: an emerging target for diseases of ageing. Nat Rev Drug Discov.

[102] Magalhães J.P., de, Wuttke D., Wood S.H., Plank M., Vora C. (2012). Genome-environment interactions that modulate aging: powerful targets for drug discovery. Pharmacol Rev.

[103] Hou Y. (2019). Ageing as a risk factor for neurodegenerative disease. Nat Rev Neurol.

[104] Baglioni C. (2016). Sleep and mental disorders: a meta-analysis of polysomnographic research. Psychol Bull.

[105•] Winsky-Sommerer R. (2019). Disturbances of sleep quality, timing and structure and their relationship with other neuropsychiatric symptoms in Alzheimer’s disease and schizophrenia: Insights from studies in patient populations and animal models. Neurosci Biobehav Rev.

[106] Dawson T.M., Golde T.E., Lagier-Tourenne C. (2018). Animal models of neurodegenerative diseases. Nat Neurosci.

[107] Woerman A.L. (2017). The importance of developing strain-specific models of neurodegenerative disease. Acta Neuropathol (Berl.).

[108] Lim A.S.P., Kowgier M., Yu L., Buchman A.S., Bennett D.A. (2013). Sleep fragmentation and the risk of incident Alzheimer’s disease and cognitive decline in older persons. Sleep.

[109] Lucey B.P. (2019). Reduced non-rapid eye movement sleep is associated with tau pathology in early Alzheimer’s disease. Sci Transl Med.

[110] Shokri-Kojori E. (2018). β-Amyloid accumulation in the human brain after one night of sleep deprivation. Proc Natl Acad Sci U S A.

[111] Vaou O.E., Lin S.H., Branson C., Auerbach S. (2018). Sleep and dementia. Curr Sleep Med Rep.

[112] Diep C. (2020). Acoustic slow wave sleep enhancement via a novel, automated device improves executive function in middle-aged men. Sleep.

[113] Garcia-Molina G. (2018). Closed-loop system to enhance slow-wave activity. J Neural Eng.

[114] Navarrete M. (2019). Examining the optimal timing for closed-loop auditory stimulation of slow-wave sleep in young and older adults. Sleep.

[115] Simor P. (2018). Lateralized rhythmic acoustic stimulation during daytime NREM sleep enhances slow waves. Sleep.

[116] Wilckens K.A., Ferrarelli F., Walker M.P., Buysse D.J. (2018). Slow-wave activity enhancement to improve cognition. Trends Neurosci.

